# Longitudinal Trends and Outcomes in Transcatheter Aortic Valve Implantation: a report from the SwissTAVI Registry

**DOI:** 10.1007/s12928-026-01310-1

**Published:** 2026-06-15

**Authors:** Thorald Stolte, Jakob Johannes Reichl, David Tueller, Felix Goetzinger, Max Wagener, Stefan Stortecky, Raban Jeger, Oliver Reuthebuch, Stefan Toggweiler, Enrico Ferrari, Peter Wenaweser, Fabian Nietlispach, Christoph Huber, Marco Roffi, Dik Heg, David Meier, Stephan Windecker, Felix Mahfoud, Stephane Noble, Thomas Nestelberger

**Affiliations:** 1 Department of Cardiology, Cardiovascular Research Institute Basel (CRIB), University Hospital Basel, University of Basel, 4, CH-4031 , Basel, Switzerland; 2 Department of Cardiology, Triemli Hospital Zurich, Zurich, Switzerland; 3 Department of Cardiology, Bern University Hospital, Inselspital, University of Bern, Bern, Switzerland; 4 Department of Cardiology, Lausanne University Hospital – CHUV, Lausanne, Switzerland; 5 Department of Cardiac Surgery, University Hospital Basel, University of Basel, Basel, Switzerland; 6 Department of Cardiology, Heart Center, Cantonal Hospital Lucerne, Lucerne, Switzerland; 7 Department of Cardiovascular Surgery, Cardiocentro Ticino Institute, Lugano, Switzerland; 8 Heart Clinic Hirslanden, Hirslanden Clinic Zurich, Zurich, Switzerland; 9 Cardiovascular Center Zurich, Hirslanden Klinik im Park, Zurich, Switzerland; 10 Division of Cardiology and Cardiovascular Surgery, University Hospital, Geneva, Switzerland; 11 Department of Clinical Research, University of Bern, Bern, Switzerland; 12 Division of Cardiology, University Hospital, Lausanne, Switzerland

**Keywords:** Aortic stenosis, Aortic valve, TAVI, Outcomes

## Abstract

The progressive expansion of indications for Transcatheter Aortic Valve Implantation (TAVI) towards lower-risk and younger populations has raised concerns about potential outcome attenuation with increasing procedural volumes. Real-world evidence addressing this concern is limited. To assess temporal trends in patient characteristics and procedural outcomes of TAVI in a large nationwide cohort. The Swiss TAVI Registry prospectively enrolled consecutive patients undergoing TAVI in Switzerland. Baseline characteristics, procedural complications and outcomes were compared across three periods: 2011-2015, 2016-2020 and 2021-2024. The primary endpoint was major adverse cardiovascular events (MACE: all-cause mortality, non-fatal myocardial infarction, and non-fatal stroke) at one year. The secondary endpoint was 30-day MACE. Among 19’452 patients, TAVI volume increased 27% annually. Patient age (82 years) and comorbidity burden remained stable, while the proportion of women increased (51% to 58%) and the median STS score decreased (4.5% to 3.3%). Considering the expansion to lower-risk patients, 1-year MACE declined significantly from 16% to 14% to 9.1% across periods (adjusted HR 0.69, 95% CI 0.62-0.78, p<0.001). Similarly, 30-day MACE decreased from 6.9% to 5.2% to 4.0% (adjusted HR 0.66, 95%CI 0.55-0.78, p<0.001). Procedural mortality declined from 2.9% to 1.2%, periprocedural myocardial infarction from 0.4% to 0.2% and stroke from 3.2% to 2.0% (all p<0.001).Risk-adjusted TAVI outcomes improved continuously despite a significant volume increase, with improvements exceeding those expected from lower-risk patients risk profiles alone.

**Supplementary Information:**

The online version contains supplementary material available at https://doi.org/10.1007/s12928-026-01310-1.

## Introduction

Over the last two decades, the management of aortic valve disease has been fundamentally transformed by transcatheter aortic valve implantation (TAVI) [1]. Initially developed as an alternative for patients with severe aortic stenosis (AS) who were deemed inoperable or at prohibitive surgical risk, TAVI has rapidly evolved into the standard of care for patients with severe tricuspid AS. Consequently, its application has been rapidly expanded to patients with intermediate- and low-risk [2]. Large randomized trials across the entire risk spectrum have consistently demonstrated non-inferior – and in selected populations superior – outcomes compared with surgical aortic valve replacement (SAVR) [3–6]. As a result, current European Society of Cardiology (ESC) guidelines now recommend transfemoral TAVI as the treatment of choice for severe tricuspid AS in patients aged 70 years and older [7]. During this period of rapid adoption, TAVI technology, patient selection, implantation techniques, and operators’ experience have evolved substantially. Advances in valve design, delivery systems, and periprocedural management have contributed to lower rates of vascular complications, paravalvular regurgitation, and early adverse events [8–10]. At the same time, the TAVI population has become increasingly diverse as indications broaden and treatment is offered to younger and lower-risk patients. Despite robust evidence from randomized trials, important gaps remain in understanding how these advances have translated into clinical practice. Although several national and international registries have reported outcomes following TAVI, most have focused on aggregate cohort outcomes or relatively short observation periods, often without a comprehensive longitudinal assessment of changes in patient characteristics, procedural strategies, and clinical outcomes over time [11–13].

This study aimed to analyze temporal trends and changes in patient demographics, procedural characteristics and short- and long-term outcomes among all patients undergoing TAVI in Switzerland over a 14-year period, providing a comprehensive real-world perspective on evolution and current state of TAVI after more than two decades of clinical implementation.

## Methods

All patients undergoing TAVI in Switzerland are prospectively enrolled in the nationwide SwissTAVI Registry as mandated by Swiss health authorities (NCT01368250). Several analyses from the registry have been published [14–16]. The study protocol was approved by cantonal ethics committees and institutional review boards of all participating centers, and all patients provided written informed consent for participation and prospective follow-up. The research conducted for this manuscript complied with the principles of the Declaration of Helsinki.

### Data collection and study endpoints

Baseline characteristics, procedural details, and follow-up information were prospectively recorded in a centralized, web-based database. Clinical follow-up was obtained through standardized interviews, reports from referring physicians, and hospital discharge summaries at 30 days and annually thereafter. All adverse events were systematically adjudicated by a clinical event committee. Clinical endpoints were adjudicated according to the Valve Academic Research Consortium (VARC) II criteria [17]. The STS Predicted Risk of Mortality (STS-PROM) Score was calculated for each patient using the version of the STS risk model current at the time of the procedure, with model updates implemented as released by the Society of Thoracic Surgeons [18–20].

All patients who underwent TAVI between 2011 and 2024 at participating centers of the SwissTAVI registry with available data were included in the analysis. Patients were then categorized into three groups based on the year of intervention: 2011–2015 (5 years), 2016–2020 (5 years), and 2021–2024 (4 years). The primary outcome of the study was a combined endpoint of major adverse cardiovascular events (MACE), including all-cause mortality, non-fatal myocardial infarction (MI), and non-fatal stroke at one year. Secondary outcomes included MACE at 30 days and the different components of the primary MACE endpoint at 30 days and one year. Additionally, procedural complications, occurring within 24 h of the intervention, were assessed.

### Statistical analysis

Categorical variables are shown as frequencies and percentages. Continuous variables are presented as median values and the 25th and 75th percentiles. Thirty-day and one-year outcomes were compared between patient groups using Cox’s regressions with Wald chi-square tests and adjusted for age, sex, left ventricular ejection fraction (LVEF), estimated glomerular filtration rate (eGFR), diabetes and history of cardiac surgery based on previous literature and matter-based knowledge. Procedural variables were specifically not adjusted for to reflect the changing procedural standards across the study period. Outcomes are visualized as Kaplan-Meier curves and hazard Ratios (HR) with 95% Confidence Intervals (CI). Temporal trends are presented in stacked bar graphs, sorted stream graphs, and smoothed line graphs with 95% CIs. Smoothing was performed using binomial generalized linear models (GLM) with a logit link and cubic splines. Up to 2 interior knots were placed around the 33rd and 67th percentiles, with boundary knots placed at the minimum and maximum years. Predictions were then back transformed to obtain smoothed rates with corresponding 95% CIs. To formally assess whether rates change over time, each GLM was compared with the corresponding intercept-only (constant-rate) model using a likelihood ratio test. A two-sided p-value of less than 0.05 was considered significant. All statistical analyses were performed using R statistics version 4.5.1 (2025-06-13) (R Foundation for Statistical Computing, Vienna, Austria). The following packages were used: tidyverse, haven, tinter, wesanderson, ggplot2, ggstream, purr, scales, ggtext, patchwork, survival, survminer, and splines.

## Results

### Baseline characteristics

A total of 19,452 patients were included in the present analysis. Among these, 3311 (17.0%) underwent TAVI between 2011 and 2015, 7968 (40.9%) between 2016 and 2020, and 8173 (42.1%) between 2021 and 2024, with an average annual increase of 27%. Median age was 82 years, 54% were female, and the median Society of Thoracic Surgeons score (STS) was 3.5%. There was no difference in age over the study period (82.0 vs. 82.0 vs. 83.0 years). The proportion of women increased from 2011 to 2015 to 2021–2024 (51% vs. 52% vs. 58%), while the surgical risk decreased (STS score 4.5% vs. 3.3% vs. 3.3%). There was a high and generally consistent burden of comorbidities across the years. Severe AS remained the most common indication for TAVI (92%). Over the years, indications beyond severe AS have increased, with more patients undergoing TAVI for degeneration of surgical bioprostheses (3.3% vs. 4.2% vs. 6.6%), failed TAVIs (0.2% vs. 0.2% vs. 0.6%), or for severe aortic regurgitation (0.7% vs. 0.7% vs. 0.8%). Baseline characteristics are summarized in Tables 1 and 2, and **Supplemental Fig. 1**.

**Table 1:** Baseline characteristics of patients undergoing transcatheter aortic valve implantation between 2011–2015, 2016–2020 and 2021–2024

Variable	*N*	Overall *N* = 19,452^*1*^	2011-2015 *N* = 3,311^*1*^	2016-2020 *N* = 7,968^*1*^	2021-2024 *N* = 8,173^*1*^
Basic characteristics					
Female, n (%)	19,452	10,536 (54%)	1,675 (51%)	4,152 (52%)	4,709 (58%)
Age, years	19,452	82 (78, 86)	83 (79, 86)	82 (78, 86)	82 (78, 86)
Body mass index, kg/m^2	19,434	26.1 (23.3, 29.4)	25.9 (23.1, 29.4)	26.2 (23.4, 29.6)	26.0 (23.2, 29.4)
STS Score, %	19,434	3.5 (2.2, 5.5)	4.5 (3.0, 7.1)	3.3 (2.1, 5.1)	3.3 (2.0, 5.2)
**Preconditions**					
Diabetes, n (%)	19,450	5,166 (27%)	842 (25%)	2,067 (26%)	2,257 (28%)
Dyslipidaemia, n (%)	19,449	11,380 (59%)	1,745 (53%)	4,436 (56%)	5,199 (64%)
Hypertension, n (%)	19,447	15,668 (81%)	2,629 (79%)	6,377 (80%)	6,662 (82%)
Peripheral artery disease, n (%)	19,449	3,045 (16%)	576 (17%)	1,206 (15%)	1,263 (15%)
Coronary artery disease, n (%)	19,450	10,678 (55%)	1,903 (57%)	4,465 (56%)	4,310 (53%)
Prior Myocardial infarction, n (%)	19,450	2,433 (13%)	474 (14%)	938 (12%)	1,021 (12%)
eGFR (Cockroft-Gault), mL/min	19,333	51 (38, 67)	49 (37, 63)	51 (38, 67)	53 (39, 68)
**Previous interventions**					
Any Heart Surgery	19,450	2,618 (13%)	508 (15%)	1,033 (13%)	1,077 (13%)
Surgical Aortic Valve Replacement, n (%)	19,451	71 (0.4%)	19 (0.6%)	21 (0.3%)	31 (0.4%)
Coronary Artery Bypass Grafting, n (%)	19,452	1,844 (9.5%)	403 (12%)	759 (9.5%)	682 (8.3%)
Prior pacemaker, n (%)	19,450	1,880 (9.7%)	347 (10%)	732 (9.2%)	801 (9.8%)
Percutaneous Coronary Intervention, n (%)	19,450	5,427 (28%)	914 (28%)	2,266 (28%)	2,247 (27%)
Prior TAVI, n (%)	19,452	69 (0.4%)	6 (0.2%)	15 (0.2%)	48 (0.6%)
**ECG and Echocardiography**					
Baseline Rhythm, n (%)	18,514				
Sinus		14,168 (77%)	2,341 (73%)	5,910 (77%)	5,917 (78%)
Atrial fibrillation		3,132 (17%)	632 (20%)	1,333 (17%)	1,167 (15%)
Paced rhythm		1,053 (5.7%)	199 (6.2%)	416 (5.4%)	438 (5.8%)
Other		161 (0.9%)	30 (0.9%)	53 (0.7%)	78 (1.0%)
LVEF, %	17,766	60 (50, 65)	60 (46, 65)	60 (50, 65)	60 (50, 65)
Aortic valve mean gradient, mmHg	17,681	40 (30, 50)	41 (31, 52)	40 (30, 49)	40 (29, 49)
Aortic valve area, cm2	16,797	0.80 (0.60, 0.90)	0.70 (0.60, 0.90)	0.80 (0.60, 0.90)	0.80 (0.65, 0.90)
^*1*^ n (%); Median (Q1, Q3)					

Abbreviations: eGFR – estimated glomerular filtration rate, LVEF – left ventricular ejection fraction, TAVI – transcatheter aortic valve implantation, STS – Society of Thoracic Surgeons

**Table 2:** Procedural characteristics of patients undergoing transcatheter aortic valve implantation between 2011–2015, 2016–2020 and 2021–2024

Variable	*N*	Overall *N* = 19,452^*1*^	2011-2015 *N* = 3,311^*1*^	2016-2020 *N* = 7,968^*1*^	2021-2024 *N* = 8,173^*1*^
Procedural Characteristics					
Indication for TAVI, n (%)	19,452				
Severe Aortic Stenosis		18,249 (94%)	3,173 (96%)	7,560 (95%)	7,516 (92%)
Severe stenosis of bioprosthesis		709 (3.6%)	73 (2.2%)	227 (2.8%)	409 (5.0%)
Severe Aortic Regurgitation		148 (0.8%)	24 (0.7%)	58 (0.7%)	66 (0.8%)
Severe regurgitation of bioprosthesis		277 (1.4%)	35 (1.1%)	108 (1.4%)	134 (1.6%)
Degenerative TAVI		69 (0.4%)	6 (0.2%)	15 (0.2%)	48 (0.6%)
Device type, n (%)	19,376				
Balloon-expandable		9,780 (50%)	1,693 (51%)	3,782 (48%)	4,305 (53%)
Self-expandable		9,596 (50%)	1,609 (49%)	4,177 (52%)	3,810 (47%)
Device size, mm	19,425	26.00 (25.00, 29.00)	26.00 (26.00, 29.00)	26.00 (25.00, 29.00)	26.00 (25.00, 29.00)
Procedural access, n (%)	19,449				
Transfemoral		17,925 (92%)	2,705 (82%)	7,342 (92%)	7,878 (96%)
Transapical		696 (3.6%)	350 (11%)	246 (3.1%)	100 (1.2%)
Other		828 (4.3%)	256 (7.7%)	380 (4.8%)	192 (2.4%)
Procedure time, minutes	16,619	52 (39, 73)	66 (50, 89)	50 (37, 73)	49 (37, 65)
Contrast volume, mL	15,860	122 (86, 175)	165 (110, 230)	123 (90, 170)	110 (80, 152)
Length of hospital stay, days	19,377	7.0 (5.0, 10.0)	8.0 (7.0, 12.0)	7.0 (6.0, 10.0)	6.0 (4.0, 8.0)
**Procedural complications**					
Procedural mortality, n (%)	19,452	350 (1.8%)	96 (2.9%)	159 (2.0%)	95 (1.2%)
Periprocedural resuscitation, n (%)	18,750	253 (1.3%)	50 (1.9%)	119 (1.5%)	84 (1.0%)
Coronary occlusion, n (%)	19,450	56 (0.3%)	14 (0.4%)	22 (0.3%)	20 (0.2%)
Stroke, n (%)	19,452	470 (2.4%)	106 (3.2%)	199 (2.5%)	165 (2.0%)
New pacemaker, n (%)	19,452	2,648 (14%)	585 (18%)	1,083 (14%)	980 (12%)
Myocardial infarction, n (%)	19,452	72 (0.4%)	18 (0.5%)	37 (0.5%)	17 (0.2%)
Major vascular complication, n (%)	19,452	1,411 (7.3%)	313 (9.5%)	744 (9.3%)	354 (4.3%)
Minor vascular complication, n (%)	19,452	834 (4.3%)	193 (5.8%)	431 (5.4%)	210 (2.6%)
^*1*^ n (%); Median (Q1, Q3)					

Abbreviations: TAVI – transcatheter aortic valve implantation

**Figure:**
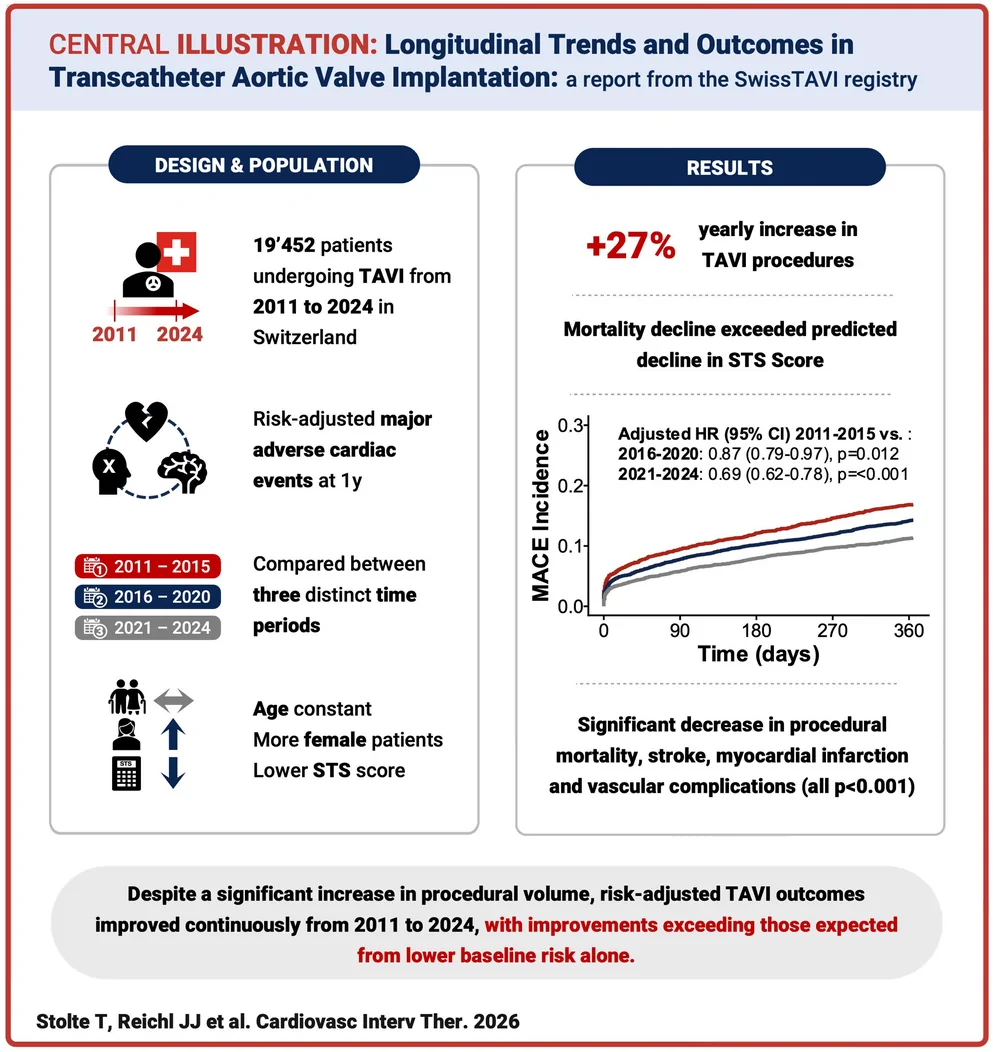
**Central illustration**: Longitudinal Trends and Outcomes in Transcatheter Aortic Valve Implantation: a report from the SwissTAVI registry. TAVI: Transcatheter aortic valve implantation, MACE: Major adverse cardiac events, HR: Hazard ratio, STS: Society of thoracic surgeons, MI: Myocardial infarction

### Procedural characteristics

**Fig. 1:**
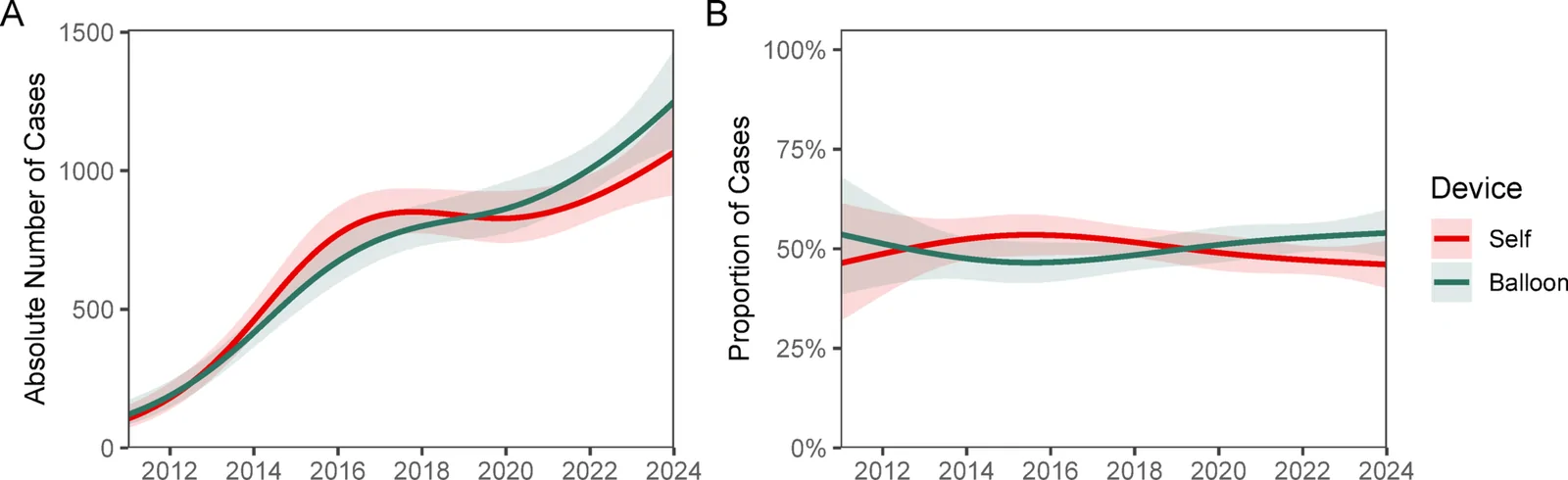
Yearly number of transcatheter aortic valve implantation procedures and respective absolute (**A**) and relative (**B**) share of balloon- and self-expandable valve systems from 2011 to 2024

**Fig. 2:**
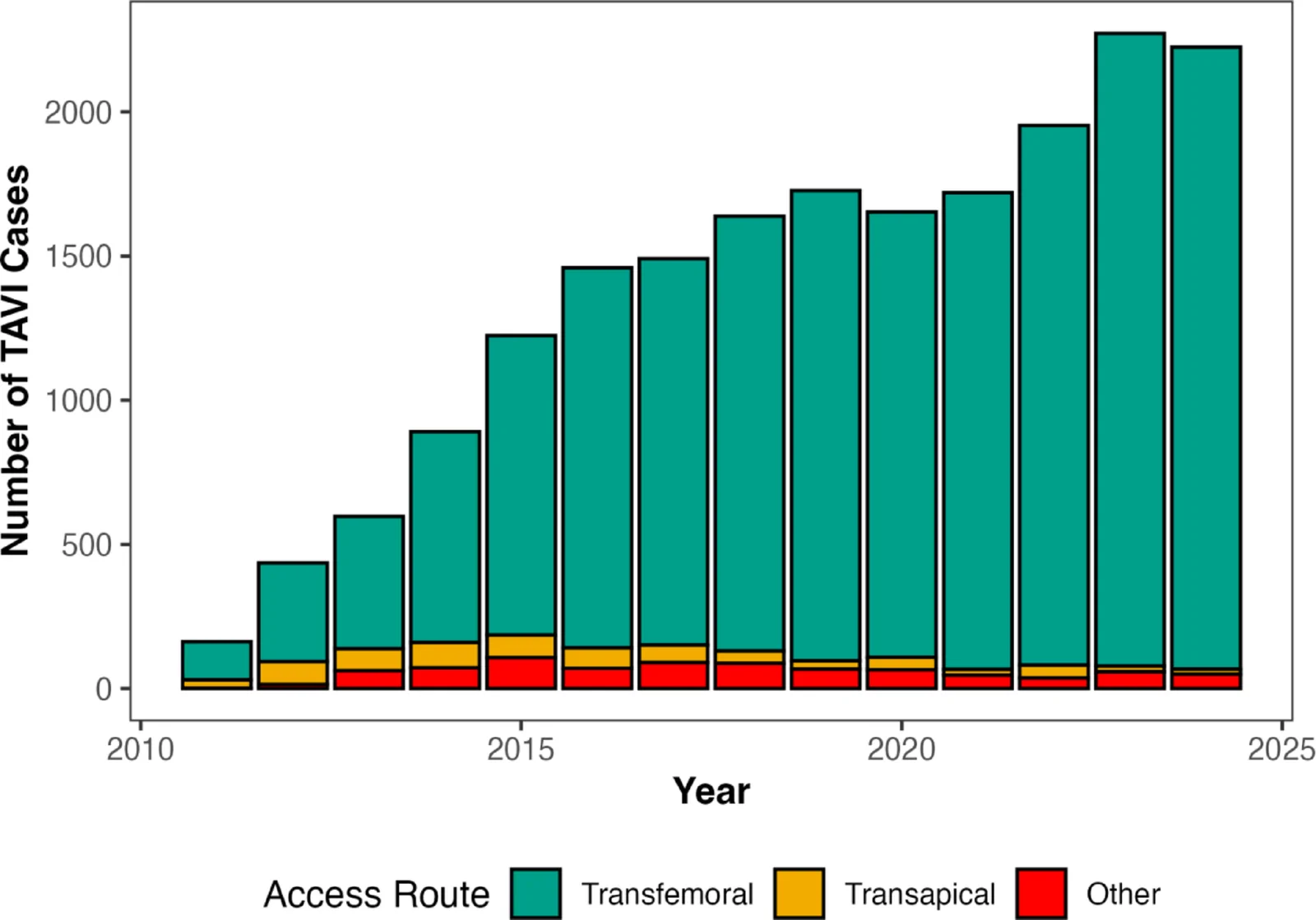
Yearly number of transcatheter aortic valve implantation procedures and respective share of transfemoral, transapical, and other access routes from 2011 to 2024.

The proportions of balloon-expandable and self-expanding transcatheter heart valves (THV) remained stable over time (51% balloon-expandable in 2011–2015 vs.53% in 2021–2024) (Fig. 1). Transfemoral access was the dominant delivery route, increasing from 82% in 2011–2015 to 95% in 2021–2024, while the rate of transapical access (11% vs. 3.1% vs. 1.2%) or other vascular access (7.7% vs. 4.8% vs. 2.4%) declined (Fig. 2). Procedure duration (66 vs. 50 vs.49 min) and in-hospital stay (8 vs. 7 vs. 6 days) declined over time. Absolute implantation numbers of different THV over time are presented in **Supplemental Fig. 2a**. Numbers for THV categorized as “other” are shown in **Supplemental Fig. 2b**.

### Procedural complications

**Fig. 3:**
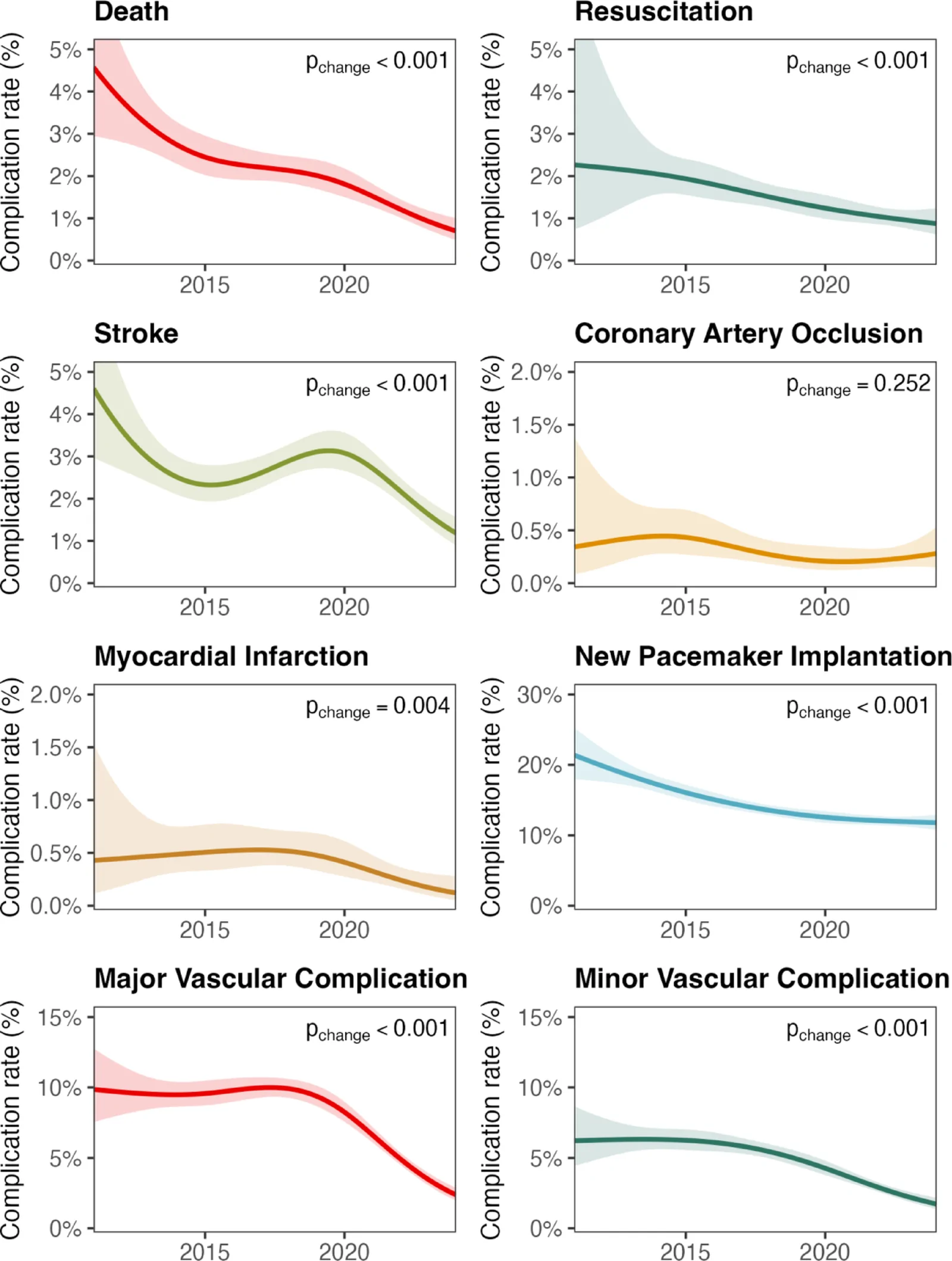
The rate of procedural complications of transcatheter aortic valve implantation over time in smoothed line graphs with 95% confidence intervals

Rates of all procedural complications (Table 2), except coronary artery occlusion, declined significantly over time (p_change_=0.252 for coronary artery occlusion, p_change_ <0.001 for all others). This was especially evident for both major (9.5% vs. 9.3% vs. 4.3%) and minor (5.8% vs. 5.4% vs. 2.6%) vascular complications, mortality (2.9% vs. 2.0% vs. 1.2%), periprocedural stroke (3.2% vs. 2.5% vs. 2.0%), and permanent pacemaker implantation (18% vs. 14% vs. 12%) (Fig. 3).

### Short- and long-term outcomes

**Fig. 4:**
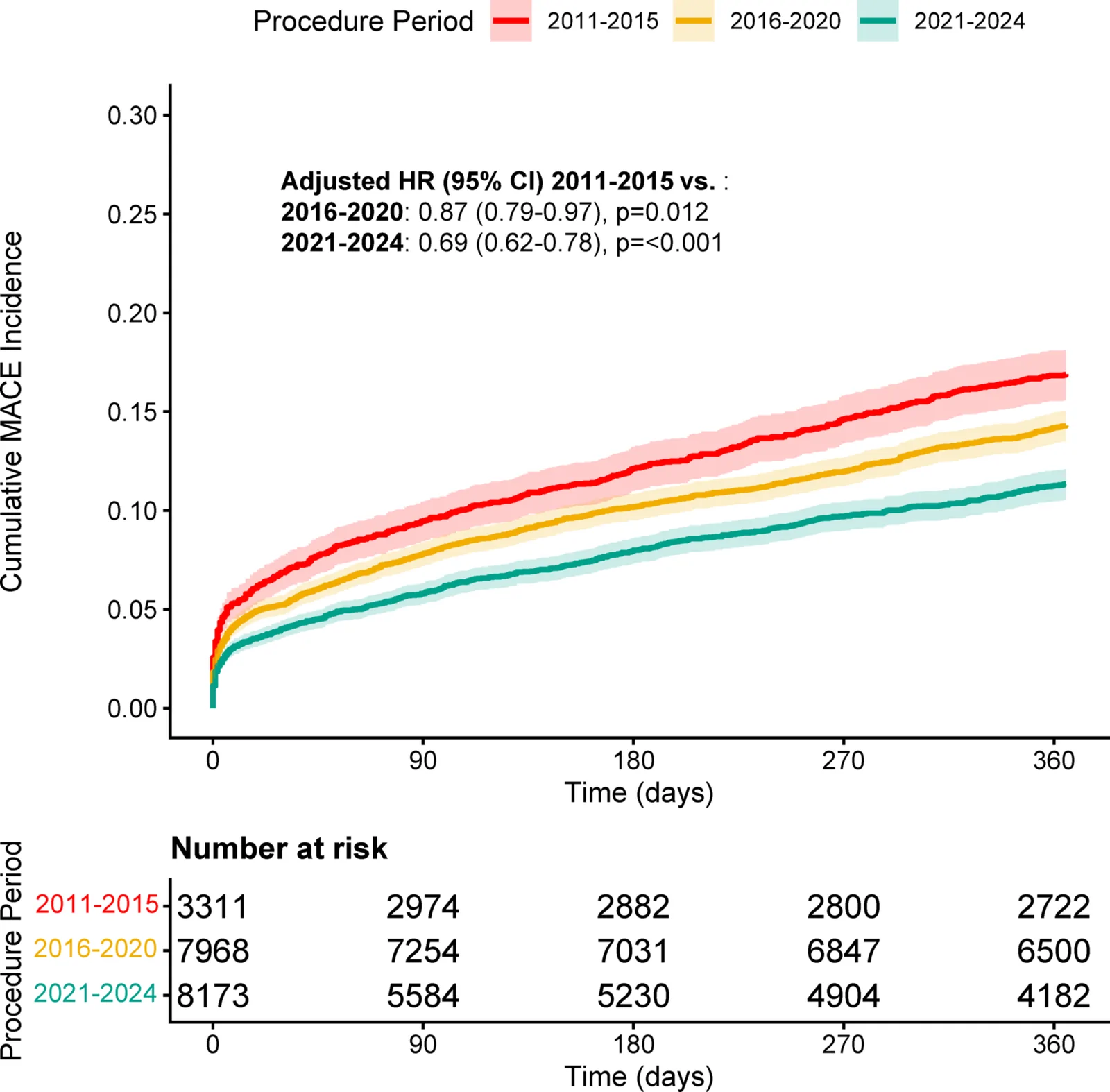

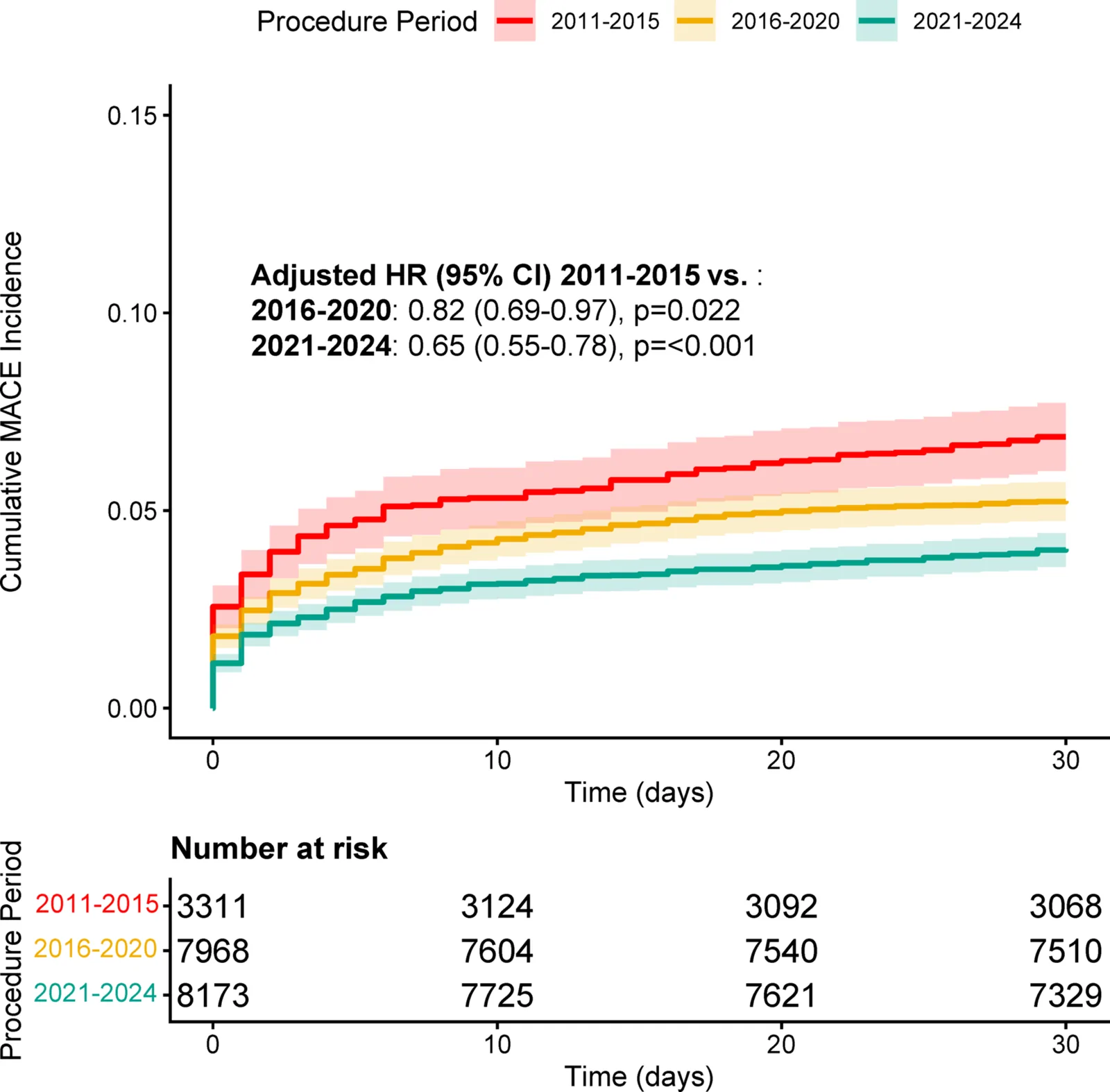
**a** The incidence of major adverse cardiac events (MACE) one year after transcatheter aortic valve implantation (TAVI) between patients undergoing TAVI 2011-2015, 2016-2020, and 2021-2024. HR: hazard ratio** b **The incidence of major adverse cardiac events (MACE) 30 days after transcatheter aortic valve implantation (TAVI) between patients undergoing TAVI 2011-2015, 2016-2020, and 2021-2024. HR: hazard ratio

**Fig. 5:**
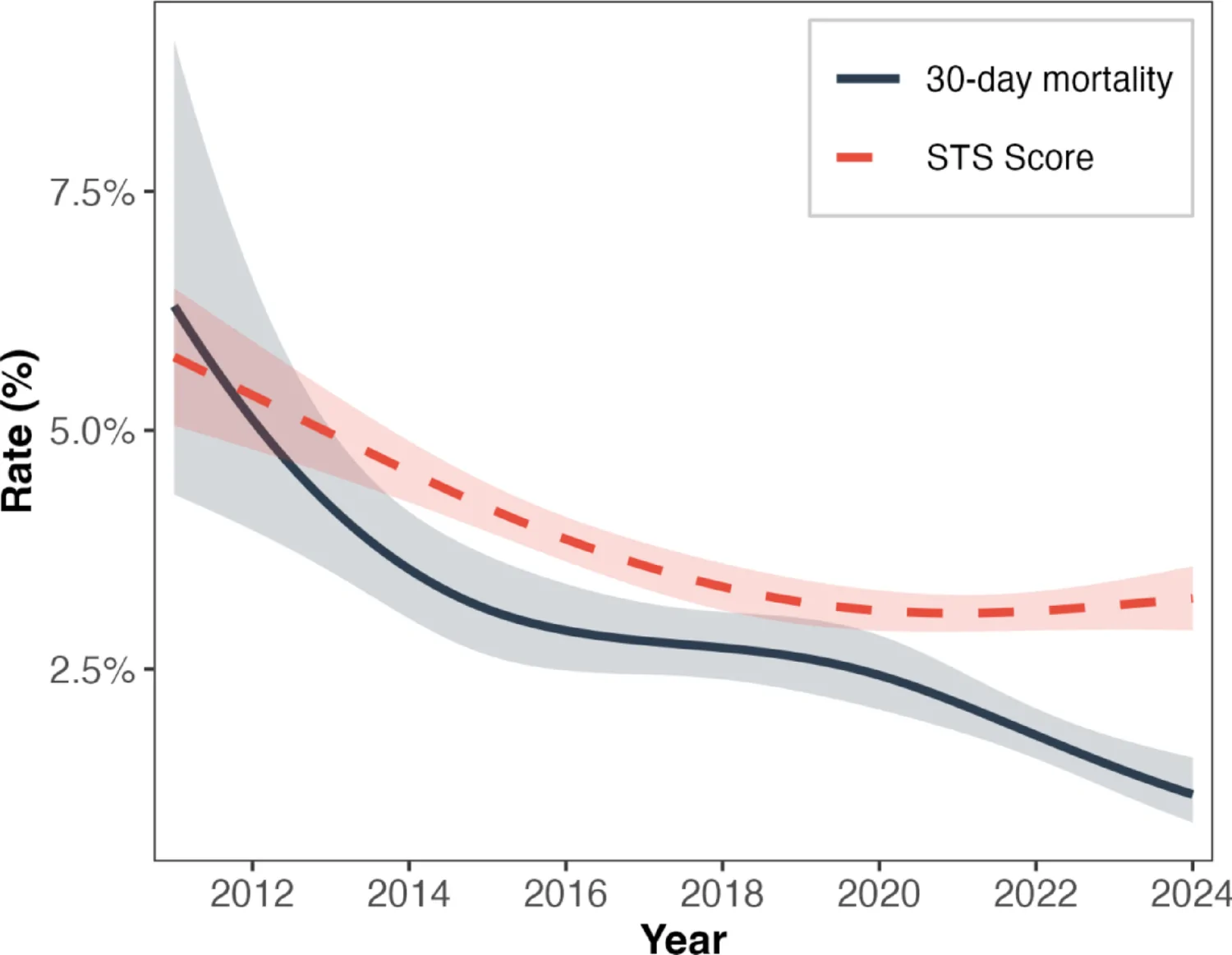
The rate of all-cause mortality at 30 days post-transcatheter aortic valve implantation and corresponding Society of Thoracic Surgeons score II over time in smoothed line graphs with 95% confidence intervals

The incidence of MACE at one year was significantly lower in procedures performed between 2021 and 2024 when compared with 2011–2015, even after adjustment for confounders listed in the methodology (16% vs. 14% vs. 9.1%, HR 0.74, 95%CI 0.66–0.82, *p* < 0.001) (Fig. 4a). This difference remained significant when the analysis was restricted to patients receiving transfemoral TAVI and those undergoing valve-in-valve TAVI. (**Supplemental Fig. 3**). MACE at 30 days declined similarly over time (6.9% vs. 5.2% vs. 4.0%, HR 0.65, 95%CI 0.55–0.78, *p* < 0.001) (Fig. 4b). The incidence of all-cause mortality (14% vs. 11% vs. 8.6%) and MI (1.5% vs. 0.9% vs. 0.6%) at one year declined over time, while there were only minor differences in stroke rates at one year (4.4% vs. 4.1% vs. 3.9%) (**Supplemental Fig. 4**). The incidence of all-cause mortality at 30 days (4.4% vs. 2.5% vs. 1.9%) was consistently lower than the corresponding calculated risk per STS-score (4.5% vs. 3.3% vs. 3.3%) (Fig. 5, **Central illustration**).

## Discussion

In this large nationwide registry study including 19,452 patients undergoing TAVI over the last 14 years, we investigated temporal trends in patient and procedural characteristics, and clinical outcomes. Our study contributes to the growing body of evidence in the field of TAVI, particularly as the number of younger and lower-risk patients treated worldwide steadily increases.

We report the following main findings. **First**, the number of TAVI procedures increased significantly from 2011 to 2024, with an average annual increase of 27%. **Second**, while overall procedural risk declined, median age remained unchanged, indicating that despite the growing number of low-risk patients, elderly patients remained the main patient population, likely reflecting reimbursement and cost-coverage restrictions. **Third**, TAVI outcomes have improved markedly, with a significant 31% relative reduction in one-year MACE and 34% relative reduction in 30-day MACE. **Fourth**, the TAVI procedure became safer, with most procedural complications and short-term outcomes, including mortality, permanent pacemaker implantation, periprocedural stroke, and vascular complications declining significantly, and to a greater extent than would be expected from the reduction in calculated baseline risk alone.

Based on the results from large, randomized trials such as the PARTNER, Covevalve, and Evolut trials, the indication for TAVI has expanded from high-risk patients to patients with intermediate to low risk [3–5, 21]. The expansion of indications, combined with an aging population and improvements in procedural techniques, device technology, and operators’ experience, has led to a 27% annual increase in TAVI volume from 2011 to 2024. While the PARTNER-IA trial enrolled patients with a mean age of 83 years and a mean STS score of 11.8%, the PARTNER III trial enrolled patients with a mean age of 73 years and a mean STS score of 1.9% [3, 5]. Although the overall surgical risk declined over time in our study, this was not reflected by a significant decrease in age or in the prevalence of common comorbidities such as hypertension, CAD, or prior MI. These findings contrast with those from the STS/ACC TVT Registry, which reported declines both in age and cardiovascular or non-cardiovascular comorbidities over time [12, 22]. These findings suggest that despite the growing number of younger and lower-risk patients, elderly and comorbid patients remained the main patient population undergoing TAVI in Switzerland, likely reflecting national reimbursement restrictions. The rising proportion of women undergoing TAVI (51% to 58%) likely reflects longer female life expectancy alongside reduced historical referral bias and guideline-driven efforts to improve equal access to the procedure [23]. As such, considering established sex-specific differences in procedural and long-term outcomes is crucial.

Importantly, MACE rates declined significantly at both 30 days and one year. The high mortality rate in the early years of TAVI has been attributed to the high surgical risk of patients, including multimorbidity, high age, and frailty, combined with first-generation devices, requiring 18–24 Fr vascular access, as well as limited operator experience [24]. Observational trials from the STS/ACC TVT Registry reported a reduction of one-year mortality from 24% to 12% from 2012 to 2018 [24, 25]. However, a stagnation of the reduction of mortality rates had been reported since 2018, and a cohort study with over 200`000 patients in the STS/ACC TVT registry showed an increase in 30-day mortality from 2019 to 2022 [12]. In contrast, our findings show a decline in both 30-day and one-year mortality, even between 2016 and 2020 and 2021–2024. The discrepancy between STS/ACC TVT Registry data and our findings may partly be explained by differences in procedural volume, which has consistently been linked to outcomes [26–29]. Additionally, the relevant decrease of most procedural complications, including vascular complications, peri-procedural stroke, and pacemaker implantation, may have contributed, as all have been associated with adverse outcomes [30–32].

Notably, the observed decline in all-cause mortality at 30 days in our cohort exceeded what would be expected from the reduction in calculated baseline risk, with predicted STS mortality remaining higher than observed mortality rates throughout the study period. This finding aligns with and extends prior literature demonstrating that the STS score, originally developed and calibrated for surgical cohorts, has limited discriminative and predictive ability when applied to TAVI populations [33–35]. The STS score has been recalibrated repeatedly since its introduction. However, since the registry employed the version that was current at the time of procedure, the growing discrepancy between predicted and observed mortality in our data cannot be explained by use of outdated calibration. This further underscores the STS score’s limited ability to capture the actual risk profile of TAVI patients, in whom frailty, anatomical factors, and procedural variables may be more relevant than typical surgical comorbidities. Although several TAVI-specific risk scores have been proposed, their external validation and clinical implementation remain limited, and reliance on surgical risk scores continues to dominate in contemporary practice [19, 36, 37]. 

While the incidence of peri-procedural stroke declined markedly over time, stroke rates at 30 days and one year exhibited a less pronounced decrease. Stroke remains one of the most feared complications of TAVI [32]. The mechanisms underlying early and late stroke differ substantially, which might explain the observed difference. While periprocedural stroke is predominantly of embolic iatrogenic origin, subacute and late stroke are increasingly attributable to patient-level factors, such as new-onset or pre-existing atrial fibrillation, subclinical leaflet thrombosis, and underlying atherosclerotic burden [38–42]. The decline in procedural stroke observed in our cohort may therefore be explained by higher operator experience, improved technologies and techniques, and advancements in preprocedural planning, all of which act on procedural rather than patient-level mechanisms. The relative stability of 30-day and 1-year stroke rates is consistent with the dominant role of patient substrate at these later time points, which procedural refinement alone is unlikely to address. However, efforts to reduce stroke rates by cerebral embolic protection devices failed to prove a significant benefit [43]. Future progress will likely require improved preprocedural identification of patients at elevated stroke risk, optimized antithrombotic strategies in the post-procedural period, and refined management of atrial fibrillation.

Finally, procedural complications, and especially vascular complications following TAVI, have declined significantly over time. Multiple factors have contributed to this improvement, including technical advancements, the development of smaller delivery systems, and a shift toward single antiplatelet therapy (SAPT) rather than dual antiplatelet therapy (DAPT) following TAVI, according to recent evidence. In early clinical TAVI trials, vascular complications were reported in approximately 15% of patients, a rate that has substantially decreased in recent years [3, 5, 44]. Moreover, the recommendations for SAPT over DAPT, introduced by both the American College of Cardiology/American Heart Association (ACC/AHA) and ESC, further influenced vascular complications [29, 45, 46]. Still, vascular complications remain unacceptably high. New approaches to vascular access have been evaluated, and radial access as a secondary access site has shown promising results in reducing vascular complications [47, 48]. These findings warrant confirmation in randomized, prospective clinical trials. The lack of a decline in coronary artery occlusion might be explained by its very low incidence, it primarily being determined by anatomical rather than procedural factors, and the observed increase in valve-in-valve procedures, which in turn have been shown to significantly increase the risk of coronary artery occlusion [49].

## Limitations

The following limitations should be considered. The classification of complications was based on VARC-2 definitions, as VARC-3 criteria were not available for all patients. This may limit the contemporaneity and comparability of our findings with other studies. The most recent year group period was one year shorter than that of the previous two groups. Furthermore, due to a lack of available data, we were unable to report additional, valve-specific outcomes like structural valve deterioration or reintervention. Despite these limitations, our study provides invaluable data collected over more than a decade across a nationwide cohort, offering important findings of trends in complications and patient outcomes following TAVI in Switzerland. The large sample size and extended observation period add robustness to the findings, highlighting the evolving outcomes landscape in TAVI patients and the impact of advances in procedural techniques and antithrombotic management.

## Conclusion

Despite a pronounced increase in TAVI procedures, short-term clinical outcomes and procedural safety have improved significantly in recent years. Ongoing investigations and continuous refinement of procedural techniques, patient selection, and procedural planning are essential to further improve patient outcomes.

## Electronic Supplementary Material

Below is the link to the electronic supplementary material.

Supplementary Material 1

Supplementary Material 2

## Abbreviations

AF: Atrial fibrillation
AS: Aortic stenosis
BMI: Body mass index
CAD: Coronary artery disease
CKD: Chronic kidney disease
CI: Confidence Intervals
CVD: Cardiovascular disease
eGFR: Estimated glomerular filtration rate
ESC: European Society of Cardiology
EuroSCORE: European System for Cardiac Operative Risk Evaluation
HR: Hazard Ratio
LVEF: Left ventricular ejection fraction
MetS: Metabolic syndrome
MI: Myocardial Infarction
NYHA: New York Heart Association
PCI: Percutaneous coronary intervention
STS-Score: Society of Thoracic Surgeons-Score
TAVI: Transcatheter aortic valve implantation
THV: Transcatheter Heart Valve
TTE: Transthoracic echocardiography
