# Supplementary material for: Longitudinal Trends and Outcomes in Transcatheter Aortic Valve Implantation: a report from the SwissTAVI Registry
Source: Cardiovasc Interv Ther. 2026 Jun 15;41(4):1124–37. doi: 10.1007/s12928-026-01310-1 (PMC13719877; doi:10.1007/s12928-026-01310-1)
Supplement: Supplementary file 2 [file 12928_2026_1310_MOESM2_ESM.docx]

**Longitudinal Trends and Outcomes in Transcatheter Aortic Valve Implantation: a report from the SwissTAVI Registry**

**SUPPLEMENTAL**

Thorald Stolte MD^1*^, Jakob Johannes Reichl MD^1*^, David Tueller MD^2^, Felix Goetzinger MD^1^, Max Wagener MD^1^, Stefan Stortecky MD MPH^3^, Raban Jeger MD^1,2^; Oliver Reuthebuch MD^5^, Stefan Toggweiler MD^6^; Enrico Ferrari MD^7^; Peter Wenaweser MD^8^; Fabian Nietlispach MD PhD^9^; Christoph Huber MD^10^; Marco Roffi MD^10^, Dik Heg PhD^11^; David Meier, MD^12^, Stephan Windecker MD^3^; Felix Mahfoud MD^1^, Stephane Noble MD^10†^, Thomas Nestelberger MD^1†^

^1^Department of Cardiology and Cardiovascular Research Institute Basel (CRIB), University Hospital Basel, University of Basel, Switzerland;

^2^ Department of Cardiology, Triemli Hospital Zurich, Zurich, Switzerland

^3^ Department of Cardiology, Inselspital, Bern University Hospital, University of Bern, Bern, Switzerland

^4^ Department of Cardiology, Lausanne University Hospital – CHUV, Lausanne, Switzerland

^5^ Department of Cardiac Surgery, University Hospital Basel, University of Basel, Basel, Switzerland

^6^ Department of Cardiology, Heart Center, Cantonal Hospital Lucerne, Lucerne, Switzerland

^7^ Department of Cardiovascular Surgery, Cardiocentro Ticino Institute, Lugano, Switzerland

^8^ Heart Clinic Hirslanden, Hirslanden Clinic Zurich, Zurich, Switzerland

^9^ Cardiovascular Center Zurich, Hirslanden Klinik im Park, Zurich, Switzerland

^10^ Division of Cardiology and Cardiovascular Surgery, University Hospital, Geneva, Switzerland

^11^ Department of Clinical Research, University of Bern, Switzerland

^12^ Division of Cardiology, University Hospital, Lausanne, Switzerland

*Both authors contributed equally to this manuscript and should be considered as first authors.

^†^Shared last authorship

**SUPPLEMENTAL FIGURES**

**Supplemental Figure 1:** The rate of comorbidities of patients undergoing transcatheter aortic valve implantation over time in smoothed line graphs with 95% confidence intervals.

**Supplemental Figure 2a:** Yearly number of transcatheter aortic valve implantation procedures and respective absolute (A) and relative (B) share of transcatheter heart valves from 2011 to 2024.

**Supplemental Figure 2b:** Yearly number of transcatheter aortic valve implantation procedures categorized as “other” in figure 2 and respective share of transcatheter heart valves from 2011 to 2024.

**Supplemental figure 3a:** The incidence of major adverse cardiac events (MACE) one year after transcatheter aortic valve implantation (TAVI) between patients undergoing transfemoral TAVI 2011-2015, 2016-2020 and 2021-2024. HR: hazard ratio

**Supplemental figure 3b:** The incidence of major adverse cardiac events (MACE) one year after transcatheter aortic valve implantation (TAVI) between patients undergoing valve-in-valve TAVI 2011-2020 and 2021-2024. HR: hazard ratio

**Supplemental figure 4:** The rate of 30-day and one-year outcomes of transcatheter aortic valve implantation over time in smoothed line graphs with 95% confidence intervals.
